# Investigations on Practical Issues in Solid Immersion Lens Based Sub-Wavelength Terahertz Imaging Technique: System Stability Verification and Interference Pattern Removal

**DOI:** 10.3390/s21216990

**Published:** 2021-10-21

**Authors:** Da-Hye Choi, Jun-Hwan Shin, Il-Min Lee, Kyung Hyun Park

**Affiliations:** Terahertz Research Section, Electronics and Telecommunications Research Institute, Daejeon 34129, Korea; jh.shin@etri.re.kr (J.-H.S.); ilminlee@etri.re.kr (I.-M.L.); khp@etri.re.kr (K.H.P.)

**Keywords:** terahertz, sub-wavelength imaging, interference pattern removal, contact-free measurement, non-destructive testing

## Abstract

Terahertz (THz) imaging techniques are attractive for a wide range of applications, such as non-destructive testing, biological sensing, and security imaging. We investigate practical issues in THz imaging systems based on a solid immersion lens (SIL). The system stability in terms of longitudinal misalignment of the SIL is experimentally verified by showing that the diffraction-limited sub-wavelength beam size (0.7 λ) is maintained as long as the SIL is axially located within the depth-of-focus (~13 λ) of the objective lens. The origin of the fringe patterns, which are undesirable but inevitable in THz imaging systems that use continuous waves, is analytically studied, and a method for minimizing the interference patterns is proposed. By combining two THz images obtained at different axial positions of the object and separated by λ/4, the interference patterns are significantly reduced, and the information hidden under the interference patterns is unveiled. The broad applicability of the proposed method is demonstrated by imaging objects with different surface profiles. Our work proves that the resolution of conventional THz imaging systems can easily be enhanced by simply inserting a SIL in front of the object with high tolerance in the longitudinal misalignment and provides a method enabling THz imaging for objects with different surface profiles.

## 1. Introduction

Terahertz (THz) imaging has proven its usefulness in various fields, such as non-destructive testing (NDT), biological sensing, and security imaging [1]. One promising application of THz technology is the contact-free inline thickness monitoring of paint films on automobiles, ships, airplanes, and other vehicles [2]. Unlike conventional thickness measurement techniques that use eddy currents and ultrasound, THz technology provides the unique characteristics of contact-free measurement and enables the monitoring of wet paint thickness [3,4]. THz technology has also proven its usefulness in the non-invasive investigation of art paintings [5]. Many optically opaque materials become transparent in the THz region, and THz waves penetrate much further into art paintings than infrared waves, which are commonly used in examinations of artworks [6]. THz imaging can reveal buried information covered by layers of paint and provide the current conservation conditions of the artwork. For the in vivo imaging of human skin, THz imaging has shown potential for applications such as scar treatment strategy evaluation and skin hydration sensing [7,8]. In these applications, contact-free measurement is also desirable though has not been extensively studied, as the measurement techniques that are presently used are affected by the pressure and occlusion effects of the imaging window onto which the imaging objects are placed [9,10].

One technical issue that plagues THz imaging is its poor spatial resolution compared with infrared and visible imaging due to its long wavelengths (1 THz = 300 μm). Efforts have been made to achieve sub-wavelength images in the THz region. Nanoscale resolved THz near-field microscopy was reported by Huber et al. in 2008 [11]. They achieved 40 nm (λ/3000) spatial resolution by detecting the THz radiation scattered from cantilevered atomic force microscope (AFM) tips. The diffraction limit can be overcome with this technique, as the spatial resolution is mainly determined by the tip size [12,13]. Although the above-mentioned techniques have superior spatial resolution, they suffer from intensity loss because they use the weak scattered radiation from the tips. Therefore, powerful THz sources and/or sensitive detectors are required. In addition, a complex setup is required to precisely control the sub-wavelength tips. Another type of sub-wavelength THz imaging system utilizes low-loss mesoscale dielectric objects of various geometries as sub-wavelength focusing lenses [14,15]. With this technique, spatial resolutions ranging from 0.3 to 0.55 λ are obtained. 

In addition to the above-mentioned techniques, a sub-wavelength imaging technique based on a solid immersion lens (SIL) is intriguing. The SIL-based imaging technique utilizes an additional high refractive index lens between the objective lens and the object to obtain more confined electromagnetic waves near the original focal plane formed by the objective lens [16]. This technique has been applied for several applications, such as optical data storage [17] and super-resolution fluorescence microscopy under cryogenic conditions [18]. Later, this technique was applied to THz regions [19,20,21,22]. A high-resistivity float-zone (HRFZ) silicon (Si) lens is suitable for use as the SIL in a THz imaging system, as the HRFZ Si has high refractive index, low absorption coefficient, and low dispersion in the THz region [23]. With this technique, spatial resolutions ranging from 0.15 to 0.35 λ have been reported [21,22]. 

When the SIL is inserted between the objective lens and the sample, understanding the system stability in terms of the longitudinal misalignment of the SIL is critical, as the focus shift from SIL misalignment would defocus the sharply converging beam on the SIL-air interface. The defocus can make the beam return to the original size obtained in the imaging system without the SIL [19]. However, the stability of a system affected by SIL misalignment and the impact of misalignment on imaging quality have not yet been experimentally characterized. In this study, we experimentally verify the stability of the system in terms of the longitudinal misalignment of the SIL. We experimentally demonstrate that the diffraction-limited sub-wavelength beam size (0.7 λ) is maintained as long as the Si lens is axially located within the depth-of-focus (DoF) (~13 λ) of the objective lens.

Although soft biological tissues have been successfully imaged with a THz SIL imaging system utilizing a Si wafer on which the imaging objects are placed [21], air gaps between the Si wafer and rigid objects lead to interference patterns that degrade the image quality [24]. For contact-free measurement, reflected THz waves from Si lens/air interfaces and sample surface strongly interfere, and undesirable fringes are visible in the THz images, which will be more discussed in the following sections. To image various objects with THz SIL imaging system (either contact-type [21] or contact-free measurements [22]), a method to eliminate the interference patterns applicable for imaging objects with different surface profiles is required. The origin of the fringe patterns is analytically studied, and a method for minimizing the interference patterns is proposed in this article. By virtue of this method, we minimize the undesirable, but inevitable interference patterns embedded in the THz image, and enable contact-free measurements. The broad applicability of the proposed method is demonstrated by imaging objects having different surface profiles.

## 2. Experimental System Characterization

### 2.1. Experimental Setup

**Figure 1:**
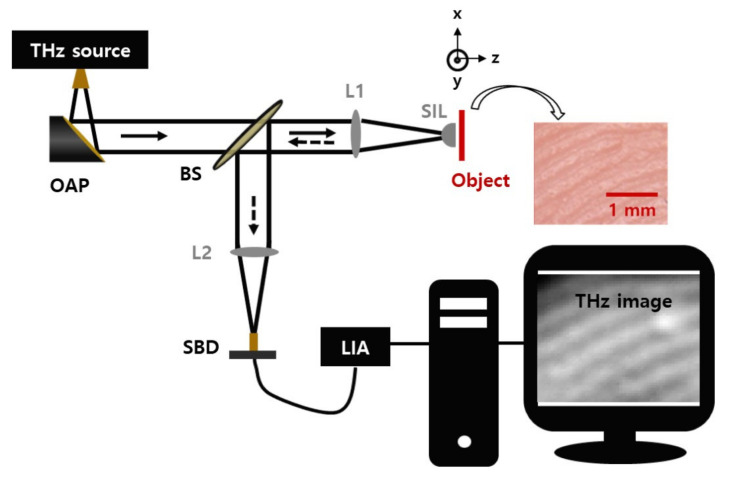
Experimental setup for THz solid immersion lens imaging. OAP—off-axis parabolic mirror; BS—beam splitter; L—lens; SBD—Schottky barrier diode; LIA—lock-in amplifier; SIL—solid immersion lens.

The experimental schematic is shown in Figure 1. For the continuous-wave THz source, we used a transmitter with amplifier-multiplier chains (VDI-Tx-S161, Virginia Diodes Inc. Charlottesville, VA, USA) at 518 GHz. The emitted THz waves from the transmitter were collimated with an off-axis parabolic mirror (OAP). The collimated wave propagates through a beam splitter (BS) made of a 100-μm-thick HRFZ Si wafer and is focused with a high-density polyethylene (HDPE) objective lens on a sample. The focal length and diameter of the lens are f = 120 mm and D = 50.8 mm, respectively. The wave reflected from the sample is reflected at the BS surface, focused with another identical HDPE lens, and detected with a Schottky barrier diode (SBD) (WR1.9ZBD, Virginia Diodes Inc.). A lock-in amplifier (LIA) was used to amplify the detected signals. For the lock-in detection, the bias voltage to the emitter was modulated with a 1 kHz square wave. 

A hemispherical Si lens with a radius of 5 mm was used as a SIL to reduce the beam size and improve the image resolution. The planar surface of the hemispherical Si lens is located at the focal plane of the objective lens. Thus, the spherical surface of the lens is concentric to the converging THz wavefront of the original wave without an Si lens [16]. The object was placed on a motorized stage (M-ILS100PP, Newport Inc. Irvine, CA, USA) driven by a controller (ESP300, Newport Inc.) for a raster scan. However, knowing the exact location of the focal plane and locating the Si lens in that plane is difficult to achieve. Therefore, understanding the experimental tolerance of the Si lens position is critical for imaging. 

### 2.2. System Stability Verification

#### 2.2.1. DoF Determination

**Figure 2:**
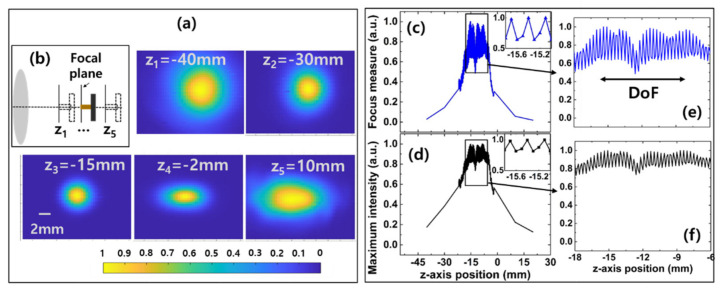
(**a**) THz beam profiles imaged at different axial positions near the focal plane without an Si lens. (**b**) A schematic showing the objective lens and the detector positions for each measurement (not to scale). (**c**) Focus measure and (**d**) normalized maximum intensity of the beam profiles. The insets of (**c**,**d**) show the zoomed-in focus measure and maximum intensity in the range from z = −15.8 to −15.0 mm, respectively. The zoomed-in (**e**) focus measure and (**f**) normalized maximum intensity in the range from z = −18 to −6 mm.

First, we define the DoF of the THz beam on the image plane without the SIL. To define DoF, beam profiles were obtained at different axial positions, and the focus measures of the images were calculated at each position accordingly. The beam profile images at different axial positions are illustrated in Figure 2a. As the THz beam propagates from the objective lens to the focal plane, the beam size decreases, and the beam shape becomes circular. After the beam passes through the focal plane, the beam size increases, and the beam shape becomes elliptical which is typical behavior of the beam after OAP. 

The focus measure functions compute the intensity difference between two adjacent pixels and are used for autofocusing and defining the DoF in various imaging systems [25,26,27,28,29]. There are many types of focus measures for autofocusing, such as the Absolute gradient, Brenner gradient, and Prewitt gradient [27,28,29]. The Prewitt gradient computes the intensity difference between two adjacent pixels along the vertical and horizontal directions, as described in Equation (1).

(1) $$\begin{array}{l} {\sum_{i}\sum_{j}\left[\begin{array}{l} I(i+1,j-1)+I(i+1,j)+I(i+1,j+1)\\ -I(i-1,j-1)-I(i-1,j)-I(i-1,j+1) \end{array}\right]}^{2}\\ +{\left[\begin{array}{l} I(i-1,j+1)+I(i,j+1)+I(i+1,j+1)\\ -I(i-1,j-1)-I(i,j-1)-I(i+1,j-1) \end{array}\right]}^{2}\;, \end{array}$$

where *I*(*i*,*j*) represents the intensity of the image with the pixel coordinate (*i*,*j*).

As shown in Figure 2c, the normalized Prewitt gradient gave three local maxima at z = −15.4, −11.3, and −8.1 mm. The Absolute and Brenner gradients also gave the same results, but the results are not shown for simplicity. The normalized maximum intensity of the beam profile image presented in Figure 2d shows similar behavior. We define the DoF as 7.3 mm (z positions between −15.4 and −8.1 mm). The zoomed-in focus measure and normalized maximum intensity in the range from z = −18 to −6 mm are shown in Figure 2e and Figure 2f, respectively. The value is consistent with the 6.8 mm obtained using the following formula [30].

(2) $$DoF=\frac{\lambda }{4n_{0}[1-\sqrt{1-{\left(NA/n_{0}\right)}^{2}}]}\;,$$

where *λ* is the wavelength, *n*_0_ is the refractive index of air, and *NA* is the numerical aperture of the objective lens. The focus measure and normalized maximum intensity values oscillate as a function of axial positions, and this has also been reported in other THz imaging systems that use a quantum cascade laser (QCL) [31]. Considering that the period of the oscillation is approximately 300 μm, as shown in the insets of Figure 2c,d, the oscillating behavior originates from the interference between the propagating wave from the emitter and the reflected wave at the detector. As local maxima can be local minima and vice versa depending on the emitter and detector positions due to this oscillation, there can be an error of approximately 300 μm for defining the exact local maxima (minima) positions. However, the DoF is not changing. In addition, the z-axis positions where THz images are obtained did not affect the beam size and image quality in the SIL-based imaging system within the DoF.

#### 2.2.2. Experimental Verification of System Stability

The stability of the THz SIL imaging system in the case of the longitudinal and transverse misalignment of optical components has been studied using FDTD simulations [24]. The simulation results showed that the spatial resolution remains close to the theoretical sub-wavelength limits, even when the displacements are on the order of wavelength λ. 

**Figure 3:**
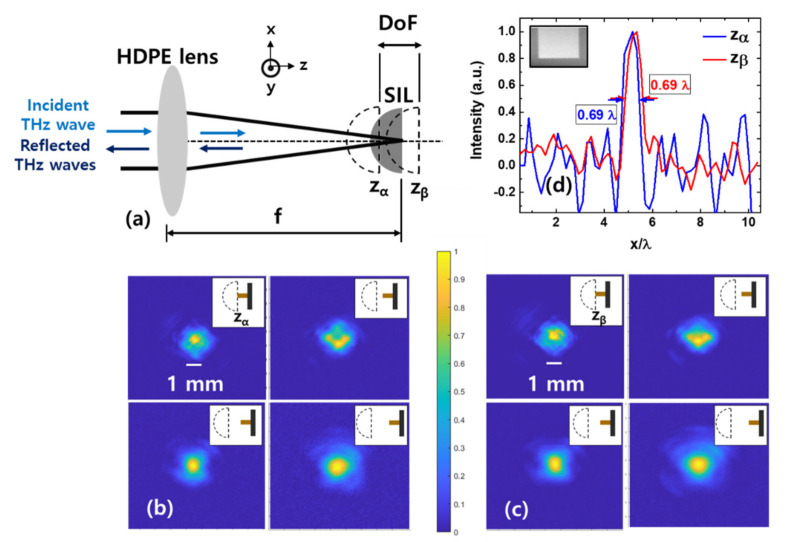
Schematic of beam focusing with the SIL and longitudinal displacements of the SIL (**a**). THz beam profiles obtained when the planar surface of the Si lens is positioned at (**b**) z = z_α_ and (**c**) z = z_β_. For each beam profile, the detector positions from the planar surface of the Si lens are 0 (top left), 2 (top right), 4 (bottom left) and 6 (bottom right) mm, respectively. Insets illustrate the Si and the detector positions for each measurement (not to scale) (**d**) THz beam size estimated at z = z_α_ and z = z_β_ by calculating the first derivative of the measured intensity of a test object (inset) featuring step-like changes in the reflectivity.

We characterized the beam properties when the Si lens is inserted at different z positions within the DoF of the objective lens to experimentally verify the stability of the optical system with respect to the longitudinal displacement of the Si lens. First, the detector was positioned to detect the maximum THz intensity for each z-position. Then, the Si lens was aligned to maximize the detected THz intensity, thereby to minimize the transverse displacement. Following this process, THz beam profiles were obtained by changing the axial position of the detector. A schematic of beam focusing with the Si lens is shown in Figure 3a. The planar surface of the hemispherical Si lens is placed at the focal plane of the HDPE lens. When the planar surface of the Si lens is placed exactly on the focal plane of the objective lens, the incident rays pass unrefracted into the Si lens and are focused on the planar surface of the Si lens [16]. As a representative data set, the beam profile images obtained when the planar surface of the Si lens is positioned at z = z_α_ and z = z_β_ (opposite ends of the DoF) are shown in Figure 3b and Figure 3c, respectively. For each beam profile set, the detector positions from the planar surface of the Si lens were 0 (top left), 2 (top right), 4 (bottom left), and 6 (bottom right) mm, respectively. We observed that the THz propagates similarly at opposite ends of the DoF in the far-field measurements. It should be noted that we cannot determine the beam size when the detector position from the Si lens is less than a wavelength, as the detector does not apply to the near-field measurements. 

To estimate the beam size (and, hence, image resolution) change due to the misalignment of the Si lens, a test object (shown in the inset of Figure 3d) that contains sharp reflectivity changes was imaged at different Si positions within the DoF of the objective lens. The image resolution was estimated from the full width at half maximum (FWHM) of the first derivative of the test object image intensity [21]. The first derivative values of the measured intensity $\left|dI/dx\right|\;$ at both ends of the DoF are shown in Figure 3d. Sub-wavelength resolution (0.69 λ) was achieved at both ends of the DoF. A theoretical image resolution limit is easily calculated using the Abbe diffraction limit for free-space optics, in which the wavelength is reduced by a factor of n [16], where n is the refractive index of the Si lens. The theoretical limit was calculated to 0.70 λ, showing that the imaging resolution of our system reached the theoretical limit. Note that the beam size did not change as we changed the distance between the Si lens and the test object up to the wavelength scale. It can also be noted that an objective lens with a low NA (NA = 0.21) was used in our imaging system, unlike previously reported THz SIL imaging that use a high NA (NA = 0.64) objective lens [21,22], to investigate the adaptability of the SIL system to conventional THz imaging systems. The image resolution could easily be improved by replacing the objective lens with a high-NA objective lens at the cost of reduced stability of the imaging system. 

## 3. Contact-Free Sub-Wavelength Imaging

### 3.1. Imaging Examples and Limitations by Interference

**Figure 4:**
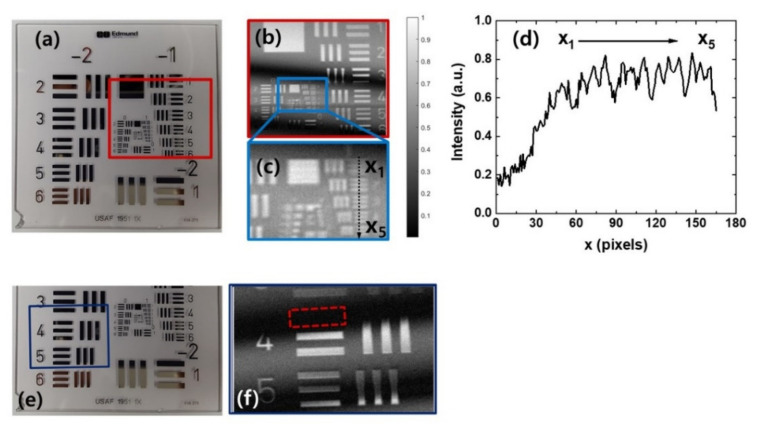
(**a**,**e**) Photographs and (**b**,**f**) THz images of the United States Air Force (USAF) resolution target. (**c**) Zoomed-in image of (**b**). (**d**) The intensity profile along the black dashed line in (**c**).

We performed the contact-free THz imaging of several objects having sub-wavelength structures. The distance between the Si lens and the objects was approximately 100 μm unless otherwise stated, and the object position is referred to as the reference position. First, images of a United States Air Force resolution target (USAF 1951 Chart, Edmund Optics, Inc. Barrington, NJ, USA) were obtained. The THz imaging areas are indicated by the red box in Figure 4a and the dark blue box in Figure 4e. Element 5 in group 1 is (line width = 158 μm; 0.27 λ) is resolved, as shown in Figure 4b,c. The intensity profile along the dashed black line in Figure 4c is shown in Figure 4d. It should be noted that a test object with periodic structures does not directly define the spatial resolution in terms of the Abbe diffraction limit or Rayleigh criterion. To estimate the imaging resolution using a test object having periodic structures, the modulation transfer function (MTF) formalism must be used [32]. To be precise, the level of contrast should be empirically defined, and the spatial frequency of the test object imaged with the defined contrast must be estimated. Then, the inverse spatial frequency of the imaged object would provide a measure of the spatial resolution, but the estimated resolution is difficult to compare with the imaging resolution in terms of the Abbe diffraction limit or Rayleigh criterion. 

In Figure 4b, the interference patterns are clearly visible in the image, and they degrade the image quality. Straight equispaced fringe patterns are observed, which can be generated by the interference of the THz waves reflected from the planar surface of the Si lens and the object plane [33]. The situation is more problematic when the structures to be imaged are under the interference patterns as shown in Figure 4f. Even though the straight equispaced fringes can be eliminated by techniques such as frequency domain filtering and inverse image processing [33], a more generalized technique is required because the shape of the interference patterns depends on the surface profiles of the objects.

**Figure 5:**
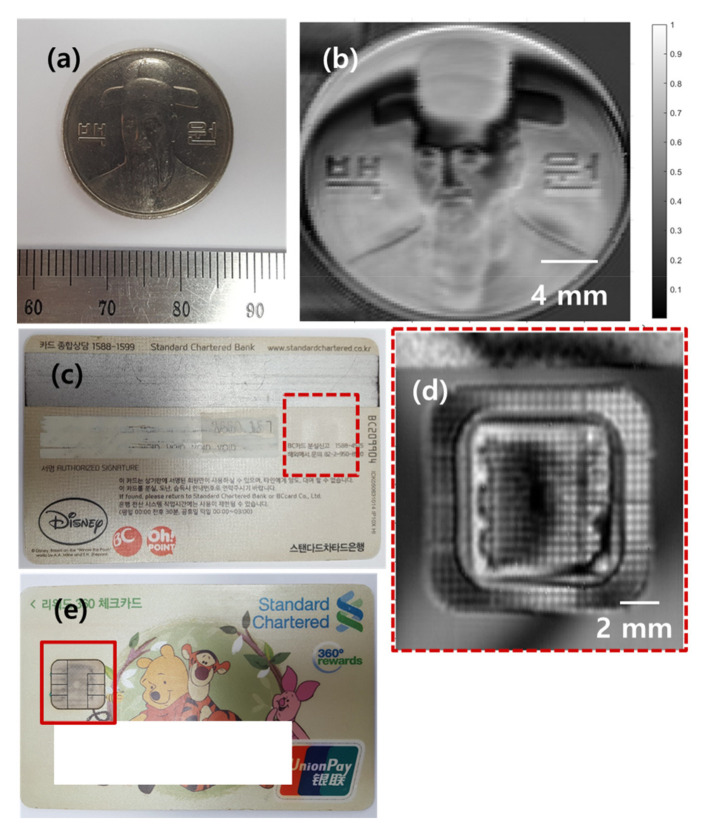
(**a**,**c**) Photographs and (**b**,**d**) THz images of a 100-won (백원) coin and an IC chip embedded in a debit card, respectively. (**e**) Photograph of the shadow side of the debit card.

Photographs and THz images of a metal coin and an IC chip embedded in a debit card are shown in Figure 5. Both objects with sub-wavelength structures were clearly imaged. Note that the THz image of the debit card was obtained from the backside of the card where the IC chip structures are invisible due to the plastic cover. The sub-wavelength structures of the IC chip embedded in a debit card are clearly revealed in the THz image (Figure 5d), showing the capability of contact-free NDT with sub-wavelength resolution. A photograph of the shadow side of the debit card is shown in Figure 5e. As the coin has non-flat structures, the THz image (Figure 5b) shows different interference patterns compared with the fringes observed in the THz image of the USAF target. The interference patterns become more complex when the object has a curved surface as shown in Figure 5d. 

### 3.2. Theoretical and Experimental Investigations on Interference Pattern Removal

To remove the interference pattern, the origin of the interference patterns should be characterized. Although Ning et al. studied three methods (tuning the frequency, location of the objects, and illumination angle of THz waves) to eliminate fringe patterns [34], only one sample was investigated, and the origin of the fringe patterns was not explained explicitly.

**Figure 6:**
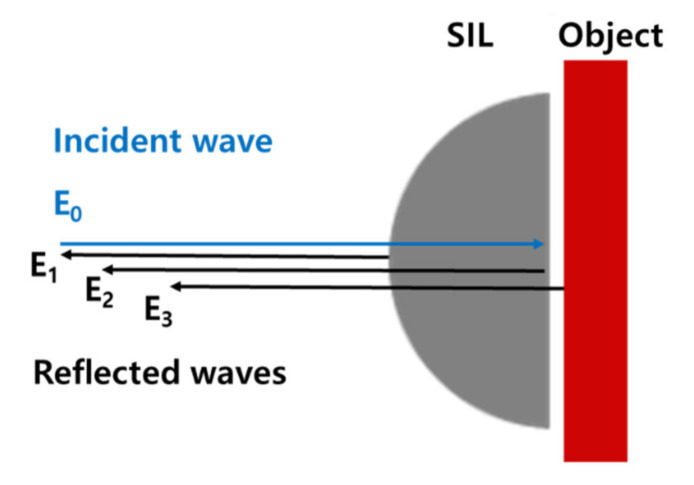
Schematic showing the location of each reflective surface in Equation (3).

In our imaging system, the Si lens provides two strong reflective surfaces, namely, the curved and planar surfaces of the Si lens, as Si is a high-refractive-index material in the THz region (n ~ 3.4) [23]. About 30% of the incident THz power is lost by reflection at a single Si lens/air interface. After the THz waves propagate through the SIL, half of the incident beam power is lost. In addition, THz waves reflected from the Si lens surfaces strongly interfere with the THz wave reflected from the sample surface, resulting in undesirable but inevitable interference patterns. When the incident electric field at the curved surface of the Si lens/air interface is *E*_0_, the measured intensity *I* can be expressed as the sum of the reflected signals from the curved surface *E*_1_, planar surface *E*_2_ of the Si lens, and sample surface *E*_3_ as shown in Figure 6.

(3) $$I={\left|E_{1}+E_{2}+E_{3}\right|}^{2},$$

where

(4) $$\begin{array}{ll} E_{1}= & E_{0}r_{01}\\ E_{2}= & E_{0}t_{01}\exp(i4\pi fRn_{1}/c)r_{01}t_{10}\\ & \times \{1-{\left[r_{10}\exp(i4\pi fRn_{1}/c)r_{10}\right]}^{MR1+1}\}\\ & /[1-r_{10}\exp(i4\pi fRn_{1}/c)r_{10}]\\ E_{3}= & E_{0}t_{01}\exp(i4\pi fRn_{1}/c)t_{10}\exp(i4\pi fL/c)r_{02}t_{01}t_{10}\\ & \times \{1-{\left[r_{02}\exp(i4\pi fL/c)r_{01}\right]}^{MR2+1}\}\\ & /[1-r_{02}\exp(i4\pi fL/c)r_{01}]. \end{array}$$

Here, *r_ij_* (*t_ij_*) is the Fresnel reflection (transmission) coefficient at the *i/j*-medium interface. Medium 0, 1 and 2 indicate air, Si lens, and sample, respectively. The symbol *f* is the frequency of the incident THz wave, *R* is the radius of the Si lens, *n*_1_ is the refractive index of the Si lens, *c* is the speed of light, *L* is the gap between the planar surface of the Si lens and the sample, and MR_1_ (MR_2_) is the number of multiple reflections inside the Si lens (the gap between the planar surface of the Si lens and the sample). During the experiment, *E*_1_ and *E*_2_ did not change, and the sum of the two terms was defined as *E_FIX_*. Then, the Equation (3) is given by

(5) $$I={\left|E_{FIX}+E_{3'}\exp(i4\pi fL/c)\right|}^{2},$$

where

(6) $$E_{3'}\;=\;E_{0}t_{01}\exp(i4\pi fRn_{1}/c)t_{10}r_{02}t_{01}t_{10}.$$

Here, we neglected the multiple reflection part in *E*_3_. This assumption is reasonable because only some of the incoming THz waves are reflected (following the Fresnel coefficient) at the Si lens-air and air-object interfaces. Moreover, the object plane is not perfectly parallel to the planar surface of the Si lens, which leads to an additional loss of detected THz waves. As the signal-to-noise ratio of the imaging system is approximately 200:1, we can easily estimate that the detected power of the THz waves experiencing multiple reflections between the planar surface of the SI lens and the object drops to the noise level. It should be noted, however, that the multiple reflections may need to be considered for highly reflective objects.

As mentioned previously, it is difficult to align the object plane perfectly parallel to the planar surface of the Si lens. This means that L varies over the image points when the sample is scanned. When the sample has a flat surface, L changes linearly, which results in sinusoidal fringe patterns over the obtained image, as experimentally shown in the previous section. When the sample has a curved surface, the fringe patterns are more complicated and difficult to describe. When the two images obtained at different axial positions separated by λ/4 are combined, the intensity of the combined image is expressed as

(7) $$\begin{array}{ll} I & \;=\;{\left|2E_{FIX}\;+\;E_{3'}\exp(i4\pi L/\lambda )\;+\;E_{3'}\exp(i4\pi L/\lambda +i\pi )\right|}^{2}\\ & \;=\;{\left|2E_{FIX}\;\right|}^{2}. \end{array}$$

Equation (7) shows that the oscillating terms resulting in interference patterns in the image are removed. 

**Figure 7:**
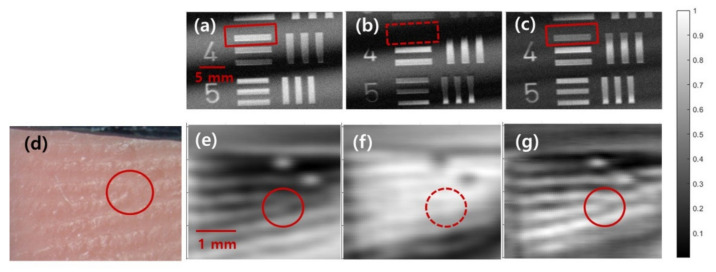
THz images of (top) the USAF target and (bottom) the dummy finger obtained at (**a**,**e**) the reference positions and at (**b**,**f**), the positions at λ/4 away from the reference positions. (**c**,**g**) The combined image of two different positions. (**d**) Photograph of the dummy finger made with dental impression material.

The above-described interference pattern removal method is applied for THz images of two objects. One has a flat surface, and the other has a complex surface profile. First, THz images of the elements of the group number −2 on the USAF target, which have a flat surface, are obtained as shown in Figure 7a–c. The THz image obtained at the reference position is shown in Figure 7a. Straight fringe patterns as predicted by the calculation were observed. The positions of the fringe patterns change as the distance between the Si lens and the sample changes. This implies that the information to be imaged can be hidden under the interference patterns depending on the sample position as indicated by the dashed red box in Figure 7b. The combined image of the two images shown in Figure 7a,b is presented in Figure 7c. The interference fringe patterns are effectively eliminated, and the image hidden under the interference patterns is revealed, as indicated by the red box in Figure 7c.

The same process was performed on the image of a dummy finger that had sub-wavelength structures and a complex surface profile, as shown in Figure 7d–f. Fingerprint patterns were copied on vinyl polysiloxane impression materials (Smart Sil, Seil Global Co., Ltd. Busan, Korea), which are used for making detailed impressions in dental clinics. The impression materials were injected on a flat surface through the mixing tip of an impression gun. Two minutes after the impression materials were injected, the mixed material was pressed with a finger. We then waited for 3 min 30 s until the impression material was completely hardened, as recommended by the supplier. All the patterns on the real finger were duplicated on the dummy finger surface, including the ridge bifurcations, prominent ridge characteristics used in fingerprint recognition techniques [35]. Fingerprint patterns are visible in the THz images. The two dots in the images are from the dust on the finger. The bright area in Figure 7e becomes a dark area in Figure 7f, and vice versa. The fringe patterns can blur or even conceal the fingerprint patterns, as indicated by the red circles in Figure 7e,f. When we combine the two images, the hidden feature is revealed, and the pattern becomes clearer as shown in Figure 7g. We demonstrate that the proposed interference elimination method is easy to adopt and can be applied regardless of the surface profile of the sample. 

Note that combining the images obtained at different frequencies can also be utilized for interference pattern removal, as implied in Equation (5). Multispectral images can be easily obtained by changing the THz source to a broadband CW source, such as UTC-PD, as the other optical components are dispersionless in the THz region [36]. This is advantageous when the sample needs to be fixed at one position, for example, in fingerprint imaging in vivo. 

## 4. Conclusions

We investigated practical issues in THz imaging systems based on a solid immersion lens (SIL). The stability of the system in terms of the longitudinal misalignment of the SIL was experimentally verified. The diffraction-limited sub-wavelength beam size was maintained as long as the Si lens was axially located within the DoF (~13 λ) of the objective lens. The results explicitly show that sub-wavelength resolution can be obtained by simply inserting a Si lens in front of the objects in a typical THz imaging system.

Then, we provided a method for minimizing undesirable but inevitable interference patterns embedded in THz images using continuous wave sources. The origin of the fringe patterns was analytically studied, and a method for removing the interference patterns was proposed. By combining two images that were obtained at different axial positions of the sample separated by λ/4, the unwanted interference patterns were significantly reduced, and important information hidden under the interference patterns was revealed, regardless of the surface profile of the sample. Our work proves that the resolution of conventional THz imaging systems can be easily enhanced by simply inserting a SIL in front of the samples with high tolerance in the longitudinal misalignment and provides a method enabling THz imaging for objects having different surface profiles. 

## Data Availability

The data presented in this study are contained within the article.
